# Lung Transplantation and the Era of the Sensitized Patient

**DOI:** 10.3389/fimmu.2021.689420

**Published:** 2021-05-26

**Authors:** Katherine A. Young, Hakim A. Ali, Kristi J. Beermann, John M. Reynolds, Laurie D. Snyder

**Affiliations:** ^1^ Division of Pulmonary, Allergy, and Critical Care Medicine, Department of Medicine, Duke University School of Medicine, Durham, NC, United States; ^2^ Department of Pharmacy, Duke University Hospital, Durham, NC, United States

**Keywords:** immunosuppression, allograft dysfunction, antibodies, rejection, lung transplant

## Abstract

Long term outcomes in lung transplant are limited by the development of chronic lung allograft dysfunction (CLAD). Within the past several decades, antibody-mediated rejection (AMR) has been recognized as a risk factor for CLAD. The presence of HLA antibodies in lung transplant candidates, “sensitized patients” may predispose patients to AMR, CLAD, and higher mortality after transplant. This review will discuss issues surrounding the sensitized patient, including mechanisms of sensitization, implications within lung transplant, and management strategies.

## Introduction

Lung transplantation has become the definitive treatment for many patients with end-stage lung disease. While there have been advances in short term survival, long-term outcomes after lung transplant are limited by the development of chronic lung allograft dysfunction (CLAD) (1). The International Society of Heart and Lung Transplant (ISHLT) defines CLAD as a substantial and persistent decline (≥20%) in measured forced expiratory volume in one second (FEV1) value from the reference (baseline) value, which is the mean of the best two postoperative FEV1 measurements (taken >3 weeks apart) (2). CLAD can present in a variety of clinical phenotypes. The two most common CLAD phenotypes include an obstructive phenotype called bronchiolitis obliterans syndrome (BOS), defined by a drop in FEV1 but initially preserved functional vital capacity (FVC) and/or preserved total lung capacity (TLC) and a restrictive phenotype called restrictive CLAD/restrictive allograft syndrome (rCLAD/RAS) which is characterized by decline in FVC and/or TLC in addition to the FEV1 decline (2). Overall, the development of CLAD portends a poor prognosis and contributes to worse survival after transplantation with the median survival being 6.5 years in the most recent era (3, 4).

With the poor prognosis of CLAD, lung transplant research has focused on mechanisms, prevention, and treatment of CLAD. One of the strongest and earliest identified risk factors for CLAD is the severity and number of acute cellular rejection episodes (5). Within the past decade, antibody-mediated rejection (AMR) or activation of humoral immunity is being recognized as a separate risk factor for poor long-term outcomes in solid organ transplantation and is considered a risk factor for CLAD in lung transplant recipients specifically (3, 4, 6, 7).

Despite early reports of patients with antibody mediated graft dysfunction, pulmonary AMR lacked a uniform definition making diagnosis and cross-center collaborative studies difficult. Therefore in 2016, ISHLT convened a working group to define pulmonary AMR (8). Alongside developing a definition and classification/grading system for AMR, the group also addressed the unique challenges of lung transplant candidates with evidence of detectable antibodies to non-self or “the sensitized” pre-transplant patient (8). This review builds on that initial report and will discuss the implications, challenges, and strategies surrounding the sensitized patient before and after lung transplant.

## Overview of AMR Mechanism in Lung Transplant

In the early 1990s, the phenomenon of antibody-mediated rejection (AMR) was first described in kidney transplant recipients (9, 10). In addition to histological changes on graft biopsy, donor-specific antibodies (DSA) were described and closely associated with graft dysfunction. The best characterized donor antibodies are specific to human leukocytes antigens (HLA) and divided into two classes (HLA Class I and II), based on their structure and function (8). Despite the wide ability to detect HLA antibodies after transplant, solid organ transplant communities have defined and responded to AMR quite differently (4, 8).

AMR in lung transplant was historically limited to hyperacute rejection, which is thought to occur when preformed DSAs bind to HLA in the donor lung. In these instances, significant and often fatal graft failure occurred within minutes to hours of transplantation and was characterized by hemorrhagic pulmonary edema, severe gas exchange limitation, and diffuse pulmonary infiltrates on imaging studies (4, 11). Subsequent identification of HLA antibodies pre-transplant and avoidance of these antigens in the donor has greatly decreased the risk of hyperacute rejection.

AMR related immune activation in the lung includes allospecific B-cells and plasma cells that produce DSAs directed against HLA on the vascular endothelium in the lung allograft. The resulting antigen-antibody complex leads to an amplified immune response or recruitment of immune cells, *via* both complement-dependent and independent pathways, and subsequent lung tissue pathology and graft dysfunction. Complement is a multifunctional system of receptors, regulators and effector molecules that may amplify both innate and adaptive immunity contributions to AMR (4, 8, 12). Notably, pulmonary AMR is different than other solid organ transplant AMR (4, 8). For instance, the lung allograft may regulate humoral responses locally (independent of secondary lymphoid organs), as well as peripherally which is contrast to other solid organs which regulate the humoral response peripherally (13).

Preliminary work in pulmonary AMR indicate complement-binding DSA are associated with worse outcomes than non complement-binding DSAs (7). DSA associated complement-independent mechanisms of allograft injury include activation of signaling cascades that leads to endothelial and smooth muscle cell proliferation, release of inflammatory cytokines/chemokines, and platelet activation. These findings suggest DSA may play a role in CLAD (4, 8). Of note, lung transplant recipients who develop DSA have a higher risk of developing chronic rejection than individuals who did not develop DSA and worse survival (3, 14). One of the strongest risk factors for post-transplant DSA is pre-transplant detectable HLA antibodies, also called allosensitization. In recent years, data from multiple centers confirmed that allosensitization prior to transplant likely increases the risk of AMR (14, 15).

## Pre-transplant Detection of HLA Antibodies- Techniques, Reporting, and Incidence

Several studies have demonstrated pre-transplant sensitization with anti-HLA antibodies are associated with decreased waitlist survival and survival after transplantation, increased ventilator days following lung transplant, higher rates of cellular rejection, development of donor-specific HLA antibodies, and bronchiolitis obliterans syndrome (BOS) (3, 16–18), however, this is not a universal finding (19). Various pre-transplant management approaches have been undertaken by the lung transplant community and largely remain institution specific (20). In addition to the potential post-transplant complications, lung transplant candidates with a high-calculated panel reactive antibody (cPRA) often have a longer waitlist time and higher risk of waitlist mortality compared with non-sensitized patients (21). To combat both the pre and post-transplant concerns, centers have employed several therapeutic approaches in an effort to lower or “desensitize” HLA antibody positive individuals prior to transplant (18).

However, many programs will decline highly sensitized lung transplant candidates (21). In a recent survey of lung transplant programs, 21.1% of programs considered a high cPRA as a contraindication to transplant, while 56.1% of programs declined offers for listed candidates who are highly sensitized on the basis of HLA antibodies to donor HLA. A minority of programs (14%) accepted offers regardless of positive virtual crossmatch or actual crossmatch (20). This variability between institutions underscores the need to better understand the effects of allosensitization on transplant related outcomes in an effort to minimize pre and post-transplant morbidity and mortality.

## Considerations for Policy Changes

Among the 3,500 transplants performed worldwide annually, approximately 60% of donors are allocated by the Lung Allocation Score (LAS) or a similar severity of disease score with a focus on maximizing transplant recipient benefit by balancing predicted mortality on the waiting list and one year survival (22, 23). While some countries have national wait lists, other countries participate in supranational allocation systems (e.g. Eurotransplant) (22). Although not accounted for in many lung allograft allocation systems, allosensitization is recognized as a barrier to transplant (21, 24).

Given the longer waitlist time and thus risk of death on the waiting list, the question has been raised on whether or not allosensitization should be weighted within the LAS or other allocation systems, though this is controversial (25). A single-center study found those with any degree of allosensitization were less likely to undergo transplant than those without HLA antibodies (17). A separate single-center study considering allosensitization as a continuous variable found allosensitization prolongs the median waiting time and significantly decreases the likelihood of transplant. This study demonstrated a direct relationship between the breadth of allosensitization (as estimated by cPRA) and waiting time, as well as an inverse relationship with the likelihood of lung transplant (21). Given these findings, consideration of allosensitization in organ allocation policies may mitigate the risk of death on the waiting list (21); but with conflicting data regarding whether or not this subset of patients experience higher mortality and complications following transplant (3, 16–19). Thus, it remains difficult to determine whether extra consideration or exceptions for sensitized patients should be made available (26). In 2022, the United States allocation through the Organ Procurement and Transplantation Network will establish a continuous distribution allocation framework and incorporate allosensitization within the new system (27).

## Pre-Transplant Sensitization and Post-transplant Outcome

As noted above, the pre-transplant sensitization is associated with variable post-transplant outcomes. As the most of these are single center, retrospective studies, they should be interpreted with caution (4). On one end of the spectrum, Bosanquet et al. showed that pre-transplant allosensitization does not adversely affect outcomes after lung transplantation such as the development of ACR, lymphocytic bronchiolitis, DSA development, CLAD, graft failure, or mortality when potentially reactive HLA are avoided in the donor by a virtual crossmatch with the recipient (19) and similar findings in CLAD were echoed by Zazueta and colleagues (28). On the other end of the spectrum, several other studies reported significant increases in mortality, acute rejection, BOS, and AMR, as well as increased post-transplant ventilator days (3, 16–18, 29). Of important note, HLA class II antibodies, especially HLA-DQ antibodies are associated with worse outcomes and may warrant special management considerations (30, 31). With the concern that allosensitized lung transplant recipients may have more acute and chronic complications after transplant, it has become a great interest to optimally manage these patients (32).

## Management Strategies: Waitlist and Allocation Considerations and Therapy

Early referral is one of the most important considerations for highly sensitized patients. Multiple societies recommend early referral to a transplant center for progressive lung disease that has a projected poor prognosis which also allows modifiable barriers to transplant (such as sensitization) to be addressed proactively to optimize candidacy or allow for early listing (33, 34). Often times lung transplant centers face the conundrum of balancing transplant urgency, which can arise as a result of late referral, with pre-transplant immunologic risk. Unfortunately, there is very little literature to guide this decision making process and is often center specific.

For sensitized patients, some centers are using strategies to potentially increase the donor pool with varying success rates. One of the first approaches is to geographically expand the donor pool using a virtual crossmatch. Rather than relying only on a prospective crossmatch for sensitized patients, which is cumbersome as it requires donor cells sent to the recipient center, a virtual crossmatch matching the donor antigens and recipient antibodies was implemented. All recipients still had a laboratory cross match, but after the transplant. Within this group of sensitized patients, the use of virtual crossmatch was associated with decreased number of days on the waitlist and decreased waitlist mortality and replicated the laboratory cross match findings (35).

Many of these management strategies aim to avoid subsequent DSA development in the recipient, as their development has been linked to adverse outcomes (3, 36). In one large single center study, DQ mismatching was an independent risk factor for the *de novo* DSA development (37). Based off this, a management strategy could consider specifically avoiding a DQ mismatch between donor and recipient, but this may not realistic based on the allocation system and recipient medical condition.

Some transplant centers have employed therapeutic approaches in an effort to lower antibodies or “desensitize” individuals prior to transplant or perioperatively (18, 38). These targeted desensitization therapies are based on antibody mediated rejection therapies. An early prospective single center study found recipients with post-transplant development of DSA who received either IVIG or combination IVIG/rituximab did not have increased risk for acute cellular rejection, lymphocytic bronchiolitis, BOS or increased mortality (39). With these encouraging results post-transplant results, several programs adopted either a pre-transplant approach or a peri-transplant desentization protocol (18, 38).

### Pre-Transplant Desensitization Approach in Lung

Using renal transplant experience, lung transplant centers have tried to desensitize pre-transplant patients (40). Appel et al. retrospectively evaluated efficacy of a peri-transplant desensitization regimen utilizing IVIG and extracorporeal immunoadsorption (ECI) in sensitized lung transplant (n=34) which found a significant reduction in ACR, however no significant difference in development of BOS or mortality following transplant (41).

### Peri-Transplant Approach

A relatively large single center study of 146 patients with known DSA or cPRA≥30% were treated with a peri-transplant desensitization protocol that included plasmapheresis, IVIG, antithymocyte globulin, and mycophenolic acid. Compared to 194 unsensitized patients, the treated sensitized recipients had significantly lower rates of acute rejection and no significant difference in spirometry, development of CLAD or 1-year graft survival (38).

In a single center study of highly sensitized patients (cPRA≥80%), 18 pre-transplant patients had an aggressive protocol that included plasmapheresis, methylprednisolone, bortezomib, rituximab, and IVIG. While there was a significant decrease in HLA antibody when measured by MFI, there was no difference in cPRA or mortality (18). Eight of 18 patients completed the protocol and went on to be transplanted.

In a recent single center report, a small group (n=5) of highly sensitized patients (cPRA≥80%) underwent a peri-transplant multimodal desensitization protocol which included plasmapheresis, rituximab, IVIG, antithymocyte globulin, carfilzomib, and belatacept. With a median follow up time of 427 days, all patients were alive with CLAD-free survival and no episodes of ACR or AMR. Treated patients had a significantly decreased waitlist time compared with historical controls. Of note, several infectious complications were noted (42).

Of recent interest in other solid organ transplants (kidney and heart) is the use of belatacept, a high affinity variant of CTLA4-IG which binds to CD80 and CD86 on antigen presenting cells thereby preventing CD28 mediated signaling critical for T cell activation and proliferation, T follicular helper cell differentiation, and cognate T/B cell interactions. Belatacept was approved for use in renal transplant recipients on the basis of two randomized controlled trials (43, 44). Several centers have reported lower incidence of DSA and superiority in constraining preexisting HLA antibody responses compared with calcineurin inhibitor-based immunosuppression (45, 46). When use in combination with desensitization strategies that focus on eliminating antibodies (plasmapheresis) and/or precursor cells responsible for antibody production (proteasome inhibitors and anti-CD20 antibodies) the hope is a more effective, durable “immune modulating” strategy that reliably and sustainably reduces HLA antibodies both before and after transplant (45–47).

Indeed there has been some promising data with the addition of belatacept to desensitization regimens in other solid organ transplant patients (47). Alishetti et al. looked at 4 highly sensitized heart patients (cPRA >99%) that underwent a multimodal desensitization protocol with a proteasome inhibitor, dexamethasome, and belatacept +/- plasmapheresis prior to heart transplant. In all four patients, desensitization with this regimen decreased the average MFI of class I and II antibodies and in most cases this response was sustained between cycles and after cessation of the proteasome inhibitor. Additionally, the chances of finding a donor to whom the recipient did not have high MFI antibodies increased markedly after desensitization (based on calculated likelihood ratios of cPRA). Of note, two infectious complications were reported which resolved with treatment (47). To date, the small cohort described in lung candidate above is the only known use of CD28 co-stimulation blockade in multimodal lung transplant desensitization protocols and larger scale trials are needed (42). The higher immunosuppressive effects are attractive in highly allosensitized patients, however safety and efficacy data are yet to be seen (48).

While some centers have reported success in regards to desensitization and transplant related outcomes, the varied protocols, heterogeneous patient populations, and relatively small sample size makes it difficult to draw conclusions regarding the role and impact of desensitization protocols especially in regards to what patients may benefit. Unfortunately, there are not randomized controlled trials that compare the clinical efficacy of different desensitization strategies.

From a logistical standpoint there is a benefit to a pre-transplant protocols that can be scheduled as opposed to peri-transplant which requires on-demand resources. However, the timing of pre-transplant may hinder the impact of protocols if the transplant occurs considerably later. Additional considerations are the off-label use of therapies which may present substantial cost to the patient or health system and can therefore be a limiting factor to accessing therapies. From a safety standpoint, potential side effects related to certain therapies should be considered in the context of the patient.

## Discussion

Pre-transplant allosensitization remains a considerable barrier in the ability to receive a transplant and avoid complications after transplant. Sensitization limits the number of donors available, thereby extending waitlist time and mortality. Efforts to expand the donor pool often includes intensive therapies that may increase the risk of morbidity and mortality. Even if a suitable donor is found, pre-transplant allosensitization increases the risk for AMR and potentially other complications including CLAD after transplant. Further research is needed in order to best manage the pre and post transplant concerns of allosensitization.

## Author Contributions

All authors contributed to the design of the manuscript and editing of the manuscript. Primary writing was done by KY. All authors contributed to the article and approved the submitted version.

## Conflict of Interest

The authors declare that the research was conducted in the absence of any commercial or financial relationships that could be construed as a potential conflict of interest.
